# A Markov State-based Quantitative Kinetic Model of Sodium Release from the Dopamine Transporter

**DOI:** 10.1038/srep40076

**Published:** 2017-01-06

**Authors:** Asghar M. Razavi, George Khelashvili, Harel Weinstein

**Affiliations:** 1 Department of Physiology and Biophysics, Weill Cornell Medical College of Cornell University, New York, NY 10065, USA; 2 Institute for Computational Biomedicine, Weill Medical College of Cornell University, New York, NY 10065, USA

## Abstract

The dopamine transporter (DAT) belongs to the neurotransmitter:sodium symporter (NSS) family of membrane proteins that are responsible for reuptake of neurotransmitters from the synaptic cleft to terminate a neuronal signal and enable subsequent neurotransmitter release from the presynaptic neuron. The release of one sodium ion from the crystallographically determined sodium binding site Na2 had been identified as an initial step in the transport cycle which prepares the transporter for substrate translocation by stabilizing an inward-open conformation. We have constructed Markov State Models (MSMs) from extensive molecular dynamics simulations of human DAT (hDAT) to explore the mechanism of this sodium release. Our results quantify the release process triggered by hydration of the Na2 site that occurs concomitantly with a conformational transition from an outward-facing to an inward-facing state of the transporter. The kinetics of the release process are computed from the MSM, and transition path theory is used to identify the most probable sodium release pathways. An intermediate state is discovered on the sodium release pathway, and the results reveal the importance of various modes of interaction of the N-terminus of hDAT in controlling the pathways of release.

The dopamine transporter (DAT) is a transmembrane (TM) secondary transporter protein belonging to the Neurotransmitter:Sodium Symporter (NSS) family, which also includes the closely related serotonin (SERT) and norepinephrine (NET) transporters^1^. The NSS are responsible for clearance of released neurotransmitters (e.g. dopamine (DA), by DAT) from the synaptic cleft through their translocation back into the presynaptic nerve termini. The function of NSS transporters in neuronal signaling implicate them in the mechanisms of action of abused psychostimulants, such as cocaine and amphetamine^2^,^3^, and in various psychiatric and neurological disorders including drug addiction, schizophrenia, and Parkinson’s disease^3^. These essential neurophysiological roles have made NSS transporters primary targets for antidepressant medications.

Breakthrough crystallographic determinations of members of the NSS family includes the bacterial homolog LeuT^4^,^5^, and most recently the structures of *Drosophila* DAT (dDAT)^6^,^7^,^8^ and SERT^9^. Structures of these transporters have been determined in complex with various substrates and ligands in the primary (S1) and secondary (S2) binding sites and with sodium ions bound in sites Na1 and Na2. They have offered an essential molecular context for the investigation of the mechanism of uphill neurotransmitter reuptake transport enabled by the coupling with the transmembrane Na^+^ gradient^1^,^10^. Our current understanding of functional mechanisms in NSS has been further shaped by the large body of structure-function studies of LeuT and other related bacterial transporters, carried out both experimentally^11^,^12^,^13^,^14^,^15^,^16^,^17^ and computationally^11^,^12^,^13^,^14^,^16^,^18^,^19^,^20^,^21^,^22^,^23^, and more recently by findings from molecular dynamics (MD) simulations of DAT^24^,^25^,^26^,^27^,^28^,^29^ and SERT^30^,^31^ constructs. Together, these studies have suggested for the transport cycle in NSS proteins an allosteric process that is consistent with the alternating access mechanism^32^ in which concerted dynamic rearrangements on the extracellular (EC) and intracellular (IC) sides of the transporter result from ion- and ligand-specific conformational rearrangements. These motions allow the transporter to transition between conformational states in which the substrate binding site is either occluded from the protein environment and does not allow water penetration either from the EC or the IC end, or alternatively exposed to the EC vestibule (in an outward-open configuration) and the IC vestibule (in an inward-open one).

One of the key mechanistic components of the early steps in the transport cycle in these transporters, which emerged from these studies, relates to the binding/release of the Na^+^ ion from the Na2 site. Indeed, studies of LeuT and other related bacterial transporters^16^,^22^,^33^ have proposed that an occupied Na2 site is essential for maintaining the transporter in the inward-closed state and that inward-opening and the subsequent substrate release process are energetically more favorable once the Na^+^ ion has left the Na2 site. Consistent with this model, MD simulations of DAT^28^ and SERT^31^ have displayed spontaneous outward-open to inward-open transition upon destabilization and inward release of the sodium from the Na2 site. From analysis of microsecond-long MD trajectories^28^ we discovered that the release of the sodium from the Na2 site (referred to hereafter as Na^+^/Na2) in the human DAT (hDAT) can be allosterically triggered by interactions of the N-terminus region of the transporter (residues 1–59) with various other regions of the transporter, and that some of these interactions are electrostatically driven and supported by the highly charged (−4e at neutral pH) PI(4,5)P_2_ (phosphatidylinositol 4,5-biphosphate) lipids in the membrane^28^,^34^,^35^.

One important aspect of the transport cycle that has not been resolved relates to the kinetics of Na^+^ ion dissociation from the Na2 site, which is essential for building kinetic models that could describe quantitatively the allosteric mechanisms involved in function of the NSS^36^. Here we describe results quantifying the rates of Na^+^ ion release from the Na2 site of hDAT from the analysis of the trajectories of extensive equilibrium atomistic MD simulations (~50 μs) using a Markov State Model (MSM)^37^. We find that starting from the transporter in the occluded state, the average time for Na^+^ ion to diffuse from the Na2 site to the IC solution is ~800 ns after the initial destabilization of the functionally important IC gates^26^,^28^,^38^ that maintain hDAT in the inward-closed state. From this analysis we also identify the existence of a meta-stable intermediate state in the Na^+^/Na2 release process, in which the Na^+^ ion that has left the Na2 site is temporarily retained within the IC vestibule of hDAT by strong interactions with residue E428. These results, presented here in the context of detailed molecular structure, provide novel insights into kinetic aspects of allosteric mechanisms in NSS transporters that had previously not been attainable from computation^36^.

## Methods

### System preparation

#### Full-length model of hDAT

The molecular model of full-length hDAT (human dopamine transporter) used in this study is the same as described and investigated earlier^28^, built from the outward-facing X-ray structure of dDAT (drosophila dopamine transporter), PDB ID code: 4M48, and including the substrate dopamine (DA) in the S1 site, as well as the two Na^+^ ions and a Cl^-^ ion in the respective ion binding sites. The complete molecular model includes the full length N- and C-termini in the conformations described in ref. 34 and is attached to the transmembrane domain as described before^28^ so that the positioning of the C-terminus closely resembled that in the dDAT X-ray structure, and the N-terminus does not to contact any residue in the TM bundle in this initial model (see ref. 28 for additional details).

#### The hDAT-membrane system

The complete hDAT model was immersed in a pre-equilibrated compositionally asymmetric bilayer membrane (see Table 1 for final lipid content, after the protein insertion), designed to resemble a neuronal cell plasma membrane^22^. This hDAT-membrane system was solvated in a 150 mM K^+^Cl^−^ TIP3P water solution with ions added to neutrality, resulting in a final atom count of ~150,000.

### Molecular Dynamics Simulations

All-atom molecular dynamics (MD) simulations were initiated with the multi-step equilibration protocol established previously^28^ and carried out with NAMD software version 2.10^39^. During this stage, the backbone of the protein was first fixed and then harmonically restrained. The solvent was initially prevented from entering the lipid-water interface. The constraints on the protein backbone were released gradually in three steps of 1 ns each, changing the force constants from 1, to 0.5, and 0.1 kcal/(mol Å^2^), respectively. This step was followed by relatively short (~20 ns) unbiased MD simulations performed with a 2 fs integration time-step and under the *NPT* ensemble (at T = 310 K), using the Particle-Mesh-Ewald (PME) method for electrostatics^40^ and the Nose-Hoover Langevin piston^41^ to control the target 1 atm pressure, with Langevin piston period and decay parameters set to 100 fs and 50 fs, respectively.

After this equilibration phase, the velocities of all the atoms in the system were reset (at T = 310 K using random number seed) and 50 independent ~1μs long unbiased MD simulations were carried out using the ACEMD software^42^, resulting in a cumulative MD simulation time of ~50 μs. The simulations with ACEMD implemented the PME method for electrostatic calculations, and were carried out according to the protocol developed at Acellera^42^ with 4 fs integration time-step and the standard mass repartitioning procedure for hydrogen atoms implemented in ACEMD. The simulations were conducted under the *NVT* ensemble (at T = 310 K), using the Langevin Thermostat with Langevin Damping Factor set to 0.1 ps^−1^.

The latest all-atom CHARMM36 force fields for proteins^43^, lipids^44^,^45^, and ions^46^ where used throughout. As recommended^45^, the total electrostatic charge on individual PI(4,5)P_2_ lipids was kept at −4*e* by protonating 50% of PI(4,5)P_2_ molecules at the “4” site and 50% at the “5” site.

### Definition of extracellular and intracellular vestibules in hDAT

To facilitate the description of large structural changes that result in water penetration into various regions of the molecule, we defined a series of local water-fillable cavities. The templates for these definitions are the extracellular (EC) and intracellular (IC) vestibules in hDAT, which had been defined before^28^ and characterized by counting water molecules. Briefly, a water molecule is considered to be in the EC vestibule if (a)-its oxygen atom is within 26 Å of the C_β_ atom of F326, but not within 5 Å of lipid atoms, and (b)-if its z-coordinate is larger than the C_β_ atom of F326 by no more than 23 Å (the z-axis is perpendicular to the membrane with its positive direction toward the EC side). Pari passu, an EC cavity is defined as a region of the EC vestibule in which water can penetrate with the oxygen atom positioned within 10 Å of the center of mass of the ligand dopamine. On the intracellular side, the algorithm assumes that a water molecule belongs to the IC vestibule if its oxygen atom is within 15 Å of the dopamine center of mass but not within 5 Å of any lipid, and if its z-directional distance to the C_β_ atom of D436 does not exceed15.5 Å. The IC vestibule is further divided into two volumes: IC channel and IC cavity. The IC channel is defined as the part of the IC vestibule that is within 12 Å of the backbone of residue T269, whereas the IC cavity is the part of the IC vestibule that lies outside the IC channel region.

### Dimensionality reduction using the “time-structure based independent component analysis” (tICA) approach

To construct a Markov model it is necessary first to reduce the dimensionality of the system in order to both remove the redundant information stored in the atomic coordinates, and provide a framework for accurate clustering of conformations (see below). For a dynamic system with multiple kinetic modes, a promising approach for accurate clustering of conformations is to separate them based on their kinetic similarity. This can be achieved by projecting the conformations of the system on its slowest reaction coordinates^47^. A metric called “time-structure based independent component analysis” (tICA) was shown recently to identify the slowest reaction coordinates of a system^47^,^48^,^49^,^50^.

Briefly, tICA is based on constructing a time-lagged covariance matrix (TLCM): ***C***_***TL***_(*τ*) = 〈***X***(*t*)***X***^*T*^(*t* + *τ*)〉 and the usual covariance matrix ***C*** = 〈***X***(*t*)***X***^*T*^(*t*)〉, where **X**(t) is the data vector at time *t, τ* is the lag-time, and the symbol 〈…〉 denotes the time average. The slowest reaction coordinates are then identified by solving the generalized eigenvalue problem: ***C***_***TL***_**V** = **CVΛ**, where **Λ** and **V** are the eigenvalue and eigenvector matrices, respectively. The eigenvectors corresponding to the largest eigenvalues identify the slowest reaction coordinates. These reaction coordinates are not unique and depend on the choice of data vector **X**. The number of elements in tICA eigenvectors is the same as the number of parameters comprising the data vector **X**. Thus, there is a one-to-one relation between parameters in **X** and elements in tICA eigenvectors. Moreover, the relative magnitude of each element in tICA eigenvectors reflects the importance of the corresponding parameter in forming a particular tICA reaction coordinate. A common practice in defining tICA parameters is to use C_α_ or C_β_ distances among all residues. While this approach is unbiased and previous studies have shown that it provides excellent tICA reaction coordinates in protein folding studies^48^,^51^,^52^, in our case this general approach couldn’t provide useful tICA reaction coordinates along which the simulation data could be discretized, because the Na^+^/Na2 release process itself is accompanied by only a limited change in secondary and tertiary structure of the protein. Indeed, projection of simulation data on such reaction coordinates yielded Gaussian distributions (data not shown) indicating that they do not contain information regarding Na^+^/Na2 release dynamics. The specific choice of the parameters for constructing tICA reaction coordinates in hDAT is described in the Results section.

### Construction of the Markov Model

Markov State Models (MSMs) have proven to be powerful tools for describing the kinetics of equilibrium processes in protein folding^53^,^54^, and in studies of functional mechanisms of various membrane proteins such as GPCRs, or activation pathways of kinases^55^,^56^. MSM validation and implementation is well documented and described in several excellent reviews^57^,^58^,^59^. Briefly, the MSM analysis of molecular dynamics is based on the idea that global conformational changes of a system can be modeled as a Markov chain, given that the transitions between different conformations are sampled at long enough time intervals so that each transition is Markovian, i.e. a transition from one state to another state is independent of the previous transitions of states. Owing to their Markovian property, the MSMs can yield information about long-timescale events from a combination of information from short time-scale events^60^.

To construct a MSM from a combination of MD trajectories, the conformational space from all trajectories is reduced by a transformation to a space defined by only a few reaction coordinates of interest. The reduced conformational space is then discretized into multiple (usually on the order of hundreds or thousands) zones (*microstates*) using automated clustering algorithms. When the microstates are defined, each conformation in the MD trajectory is assigned to the closest microstate, which produces the so-called *assignment space*. A *transition count matrix* is constructed in the next step by counting the number of transitions among all microstates. To satisfy detailed balance and local equilibrium, the transition count matrix is symmetrized using its transpose matrix or a maximum likelihood approach, as described in ref. 61. Finally, the probabilities of transitions among all microstates are calculated by normalizing the transition count matrix, which yields the *transition probability matrix* (TPM). To ensure that the behavior is Markovian, multiple TPMs are constructed for different time interval between transitions (referred to as “lag times”), and the relaxation timescales of the system are extracted by using the relation:


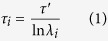


where *τ*′ is the lag-time used for building the TPM, λ_i_ is the *i*^th^ eigenvalue of the transition probability matrix and the resulting *τ_i_* is the so-called *implied timescale* corresponding to the *i*^th^ relaxation mode of the system. If the transition probability matrix is Markovian, then *τ_i_* will be independent of *τ*′ and this is the basis for the test of “Markovian behavior” for a particular choice of parameters (a specific illustration of the test is given in the Results section).

To extract information from the Markovian TPM that contains all the information about the thermodynamics and kinetics of the system, the TPM is decomposed into its eigenvalues and eigenvectors. The eigenvector corresponding to the largest eigenvalue (that is, equaling one) represents the equilibrium population of the system. The remaining eigenvectors contain kinetic information of the system and the one corresponding to the second largest eigenvalue represents the slowest relaxation dynamics of the system. In turn, the eigenvector corresponding to the third largest eigenvalue represents the second slowest dynamics of the system, and so on.

### Selection of parameters for Markov model construction

The quality of a Markov model depends on a number of parameters including the number of tICA eigenvectors used in the discretization of the conformational space, the number of microstates used in the representation of the space, and the clustering algorithm used for grouping conformations into microstates^57^,^61^. The discretization of the conformational space required for the construction of the MSM can be projected on the problem of interest by the protocol of dimensionality reduction. The number of eigenvectors used impacts the quality of MSM, as more tICA eigenvectors provide discretized states of higher resolution, but also reduce the statistical significance of the states due to finite sampling errors^62^.

To find the best combination of parameters for constructing the MSM, we used a recently introduced MSM scoring method called “generalized matrix Rayleigh quotient” (GMRQ)^63^ that is implemented in the MSMBuilder software package^61^. Briefly, this method utilizes all the simulation data to obtain the tICA space; the subsequent *assignment space* is obtained by discretization of the tICA space as described above. In the next step of the GMRQ method, the *assignment space* is divided into two sets: a training set and a test set. A TPM is then constructed for each set at a lag time that produces Markovian behavior (here we used 48 ns, see Results) and is decomposed into its eigenvectors and eigenvalues. The GMRQ algorithm then tests how well eigenvectors of the training set diagonalize the TPM of the test set, producing a score defined as:


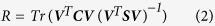


where *Tr* denotes trace of the inner matrix product in which ***V*** contains the first *n* eigenvectors of the training set TPM; ***S*** is the diagonal matrix composed of equilibrium populations of the test set (note that the equilibrium population is the first eigenvector of the TPM); and ***C*** is the inner matrix product of ***S*** and the TPM of the test set (***T***):


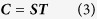


The best MSM parameters are those that yield the highest performance (highest GMRQ score) on the *test* set.

### Transition path theory (TPT) analysis

Besides providing thermodynamic and kinetic information for a system, Markov models can also be utilized to obtain the most probable pathways for a kinetic process, such as the most probable pathways for the release of Na^+^/Na2 we examine here. Transition path theory (TPT) is an elegant approach to obtain and quantify these pathways. Briefly, TPT is based on first constructing a *flux* matrix for all states of the system. A definition of the elements of the flux matrix defined in an analogous manner to the definition in ref. 64 is:


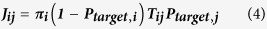


where ***π***_***i***_ is the equilibrium population of state *i, **P***_***target,i***_ is the ***P***_***target***_ for state *i* and ***T***_***ij***_ is the transition probability from state *i* to state *j. **P***_***target,i***_ is the probability of visiting the target state(s) (in our case the states with Na^+^/Na2 released) before the other states (in our case the states with Na^+^/Na2 bound), and takes values between 0 and 1.

The most probable pathways are obtained by finding those with the highest flux between the states in the flux matrix by using a graph theory algorithm, and we employed here the Dijkstra algorithm^65^ implemented in the MSMbuilder software^61^, which finds the top path. Subsequent paths are obtained by first removing the top path from the flux matrix and then repeating the algorithm. This iterative approach was used here to obtain the first 7 paths, which contribute to >60 percent of the total flux (see the Result section).

We note that whereas the MSM is built on the microstate level (with hundreds to thousands of states) in order to provide accurate timescales for the kinetics of a system, the TPT analysis, is usually performed on a *macrostate* level (on the order of a few tens of states) to facilitate the visualization of the most probable pathways. To bridge these levels, microstates are grouped together into a macrostate based on their similarity in specific aspects relevant to the information sought from the MSM analysis. Here, we used the Robust Perron Cluster Analysis (PCCA^+^)^66^ algorithm, which groups microstates based on their kinetic similarity.

## Results

### Molecular mechanism of Na^+^ release from the Na2 site of hDAT

#### PIP_2_-mediated N-terminus/IL4 interactions destabilize intracellular gates in hDAT

**Figure 1:**
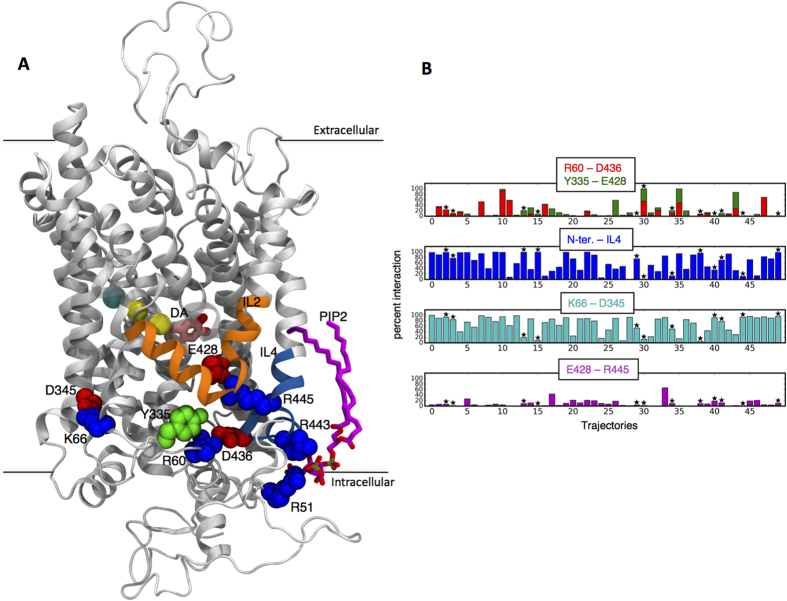
Interactions of the N-terminus with the intracellular end of hDAT in the process of Na^+^/Na2 release. (**A**) Full atomistic representation of hDAT. Residues mentioned in the analysis are shown in space-filling representation, labeled and color-coded based on their polarity, with positively charged residues in blue, polar residues in green, and the negatively charged ones in red. Na^+^ and Cl^−^ ions are shown as yellow and cyan spheres, respectively. Dopamine (DA) is shown in pink licorice. IL4 (in blue cartoon), and IL2 (in orange cartoon) are also highlighted. PIP_2_ (magenta) mediates interaction between IL4 and the N-terminus (here, R51). (**B**) The frequencies of interactions between key residues in all 50 trajectories (expressed as percent), averaged over the entire simulation time. Stars indicate the trajectories in which Na^+^/Na2 is released. The N-ter–IL4 panel represents PIP_2_ mediated interactions (within 6 Å) between various Arg and Lys residues from the N-terminus (residue 1 to 59) with Arg443 from IL4 (defined as the interaction of the same PIP2 with both Arg443 and any Arg or Lys residue of the N-terminus).

Our previous computational studies^28^ have demonstrated the mechanistic involvement of PIP_2_-mediated interactions between the N-terminus domain (N-term) and the intracellular loop 4 (IL4) of hDAT in the inward opening of the transporter and the concomitant release of the Na^+^/Na2. Here, we found the same mode of N-term/IL4 association recapitulated during the initial stages of the MD simulations we analyzed (Figure S1). Consistent with our previous results^28^, we found that the N-term/IL4 interactions resulted in hDAT conformations in which the functional intracellular gates formed by residues R60, Y335, E428, R445, and D436 (Fig. 1A)^26^,^33^, were destabilized. To expand the exploration of the conformational space, we initiated from this state of the hDAT an ensemble of 50 new, ~1 μs-long, statistically independent atomistic MD simulations. The analysis revealed that while in more than half of the trajectories the N-term could disengage from IL4 and initiate other modes of interaction with the intracellular region of hDAT, the interactions defining the intracellular gates remained nevertheless broken in the majority of the simulations (Fig. 1B). Importantly, in this ensemble of 50 simulations we observed multiple instances of spontaneous inward release of Na^+^/Na2, the Na^+^ ion from the Na2 site.

#### The isomerization of hDAT from an outward-facing to an inward-facing configuration with concomitant release of Na^+^/Na2 is determined by the extent of solvation of functional sites

Spontaneous inward release of the Na^+^/Na2 was observed in 12 of the 50 ~1 μs-long MD trajectories (Figure S2). The fastest observed release occurred after only 80 ns of MD simulation (not including the initial equilibration phase), while the slowest observed release took place after 900 ns (Figure S2). Analysis of the trajectories in which Na^+^ leaves the Na2 site reveals that the process is largely determined by the extent of water penetration from the intracellular region into the functional sites of the transporter. Indeed, increase in the distance of Na^+^/Na2 from the stable Na^+^ ion in the Na1 site is accompanied by an increase in the number of water molecules surrounding Na^+^/Na2. This is evidenced by the correlation between the water coordination number of Na^+^/Na2, and the distance of the Na^+^/Na2 from the sodium at site Na1 calculated for each of the 12 trajectories in which Na^+^/Na2 is released to the intracellular environment, as shown in Table S1.

**Figure 2:**
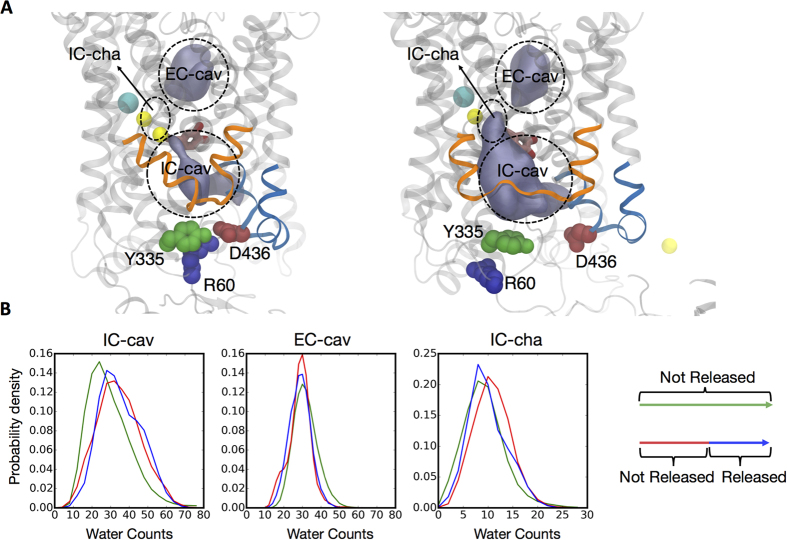
Solvation of the IC and EC cavities (IC-cav; EC-cav respectively). (**A**) Representation of the changes in water counts in EC and IC, and the breaking of the interactions among residues R60, D436, and Y335 at the intracellular end of hDAT. Note that when the interactions are broken (right panel), the water-occupied volume (in grey surface representation) is much increased both in the IC-cav and in the region including the Na2 site, identified as the IC channel (IC-cha). The color code for structural elements is the same as in Fig. 1A. (**B**) Water distribution in the intracellular cavity (IC-cav), the extracellular cavity (EC-cav), and the intracellular channel (IC-cha). Blue curves represent all the conformations in the 12 separate trajectories in which Na^+^/Na2 has been released to the intracellular environment. Red curves represent all the conformations in which Na^+^ is still in the Na2 site in these 12/50 trajectories from which Na^+^/Na2 is eventually released. Green curves represent all the conformations where sodium is in the Na2 site in the 38/50 trajectories in which Na^+^/Na2 is not released. Confidence intervals at 68% and 95% were calculated for the EC-cav distribution that has normal distribution properties (Table S2).

Concomitant with this movement of Na^+^/Na2, we observed an increase in the water count inside the intracellular (IC) vestibule (Fig. 2A), which is enabled by the open IC bridges between R60-D436, Y335-E428, and E428-R445 (see Fig. 1B). The accumulation is highest in the trajectories in which Na^+^/Na2 is released, as shown in Fig. 2 (see IC-cav). Notably, the disruption of the K66-D345 salt bridge (e.g., see replica 30 in Fig. 1B) allows water influx through another region, located between TM1a and IL3.

In extracellular (EC) vestibule the number of water molecules is seen to decrease somewhat as Na^+^/Na2 leaves (left shift of the distribution plots in Fig. 2, EC-cav), in tandem with the increase in the solvation of the IC-cav, which points to the transition of hDAT from an outward-open/inward-closed configuration to a more outward-closed/inward-open one that accompanies the release of Na^+^/Na2. The confidence intervals calculated for the water distribution in the EC-cav for the left shift to lower numbers of water molecules observed in EC-cav water distributions for the trajectories in which the Na^+^/Na2 is released, compared to the ones in which Na^+^/Na2 is not released, are presented in Table S2. This finding is consistent mechanistically with the observation in the dynamics of extracellular gates that the main extracellular gate (R85 and D476^33^) is more stable in trajectories in which Na^+^/Na2 is released compared to the other trajectories (Figure S3). The water count also increases in a somewhat deeper local region that includes the Na2 site, termed IC-cha for IC channel (Fig. 1B) (see Methods).

**Figure 3:**
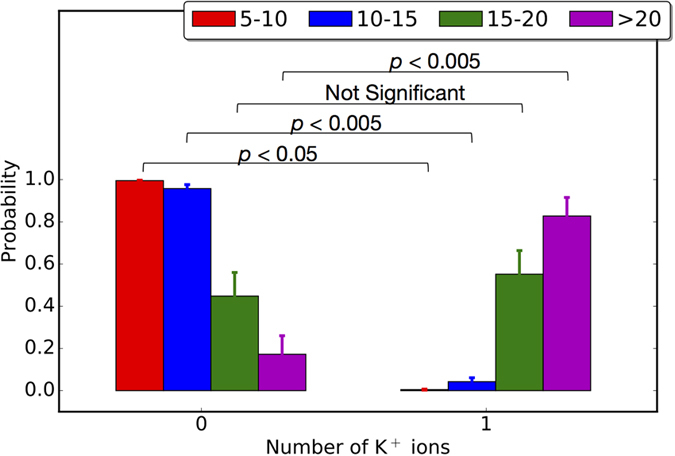
Probability of finding K^+^ ions in the binding site. The probability of finding K^+^ ions within 7 Å of D79 is compared for different states of the system in all 50 trajectories. The states are defined by the distance between the two sodiums (color code on top is in Å), and K^+^ is seen to penetrate the Na2 site only after the Na^+^/Na2 has cleared the hydration channel (i.e., Na2–Na1 distances >10 Å). The error bars are calculated using the bootstrap method with randomly selecting between 26 to 38 trajectories repeated for 100 times. *p*-values are calculated using the student T-test for the mean of variables. The *p*-values indicate the significance of probability differences between corresponding states in the presence (1 ion) and absence (0 ion) of potassium.

Notably, there is a decrease in the number of water molecules near the Na2 site after the release of the Na^+^/Na2 ion (Fig. 2B, IC-cha, compare red and blue traces), which relates to a penetration of K^+^ ions from the IC region towards the Na2 site near residue D79. The calculated probability of finding K^+^ in the Na2 site is shown in Fig. 3. This finding is intriguing in light of a known role for K^+^ ions in functional mechanism of the related SERT transporter^67^, where transitions from the inward-facing to the outward-facing state are K^+^ dependent (see Discussion).

### Kinetics of the Na^+^/Na2 release

#### The energy landscape for the release process of the Na^+^/Na2 specified with the tICA method

**Figure 4:**
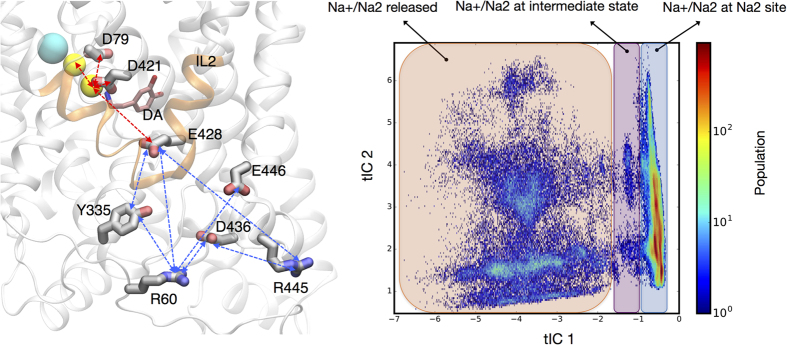
Dimensionality reduction of the entire sampled configurational space. *The left panel* depicts the two sets of parameters (distances) used for tICA construction (see also Table 2), in the structural context of the transporter. The distance parameters describing the intracellular gate dynamics are indicated with *blue arrows*; the ones used to describe the Na^+^/Na2 motion are shown with *red arrows*. Dopamine (DA) is shown in pink licorice, IL2 in orange cartoon, sodium ions as *yellow* spheres, and the chloride ion as a *cyan* sphere. *The right panel* depicts the two-dimensional tICA space. The color map represents the population of states (see color code on the right). The three major regions of this population of states are identified by their colored background: one in which the Na^+^/Na2 is still bound in the Na2 site (light blue background, on the right); the intermediate state (purple background); and configurations after the Na^+^/Na2 was released (tan color background).

The extensive conformational sampling of the system (and the Na^+^/Na2 dynamics in particular) was subjected to dimensionality reduction with the tICA method by projecting all the trajectory snapshots on tICA reaction coordinates as described in Methods. The construction of the tICA space required a definition of parameters as detailed in Methods. To obtain the specific set of parameters for constructing the tICA reaction coordinates for the release of Na^+^/Na2 (see Table 2 and Fig. 4) we used the observations of conformational changes described above, and the structural and dynamic inferences from the Na^+^/Na2 release process. The chosen parameters reflect the changes in (1) the Na^+^ ion motion as it exits through the intracellular vestibule; (2) water penetration into the IC vestibule as described by the distance among residues that form the intracellular gate (cf. Fig. 1); and (3) the water coordination number of the Na^+^/Na2 as a parameter for measuring amount of water in the binding site (cf. Fig. 2). These parameters are listed in Table 2 and represented in Fig. 4 in the context of the transporter structure.

It has been shown previously that tICA reaction coordinates are not critically dependent on the lag time used for the construction of the time-lagged covariance matrix, TLCM, described in the Methods^68^. We chose a lag time of 16 ns for the TLCM calculation, and mapped all simulation data on all 12 tICA eigenvectors. Analysis of the contributions from these eigenvectors showed that only the first three tICA eigenvectors produced distributions of the simulation data that were different from normal distribution (Figure S4), indicating that the first 3 eigenvectors are sufficient for discretization. Furthermore, statistical analysis of Markov models (see section below and Figure S6) showed that the quality of a Markov Model built from the first two tICA reaction coordinates is higher, and is thus preferable to the one built from the first three tICA reaction coordinates. Therefore we chose to use the first two tICA reaction coordinates for all subsequent analyses. The components of the tICA eigenvectors show that the first tICA reaction coordinate focuses on the dynamics of Na^+^/Na2, while the second describes dynamics of the N-terminus (Figure S5). Inspection of conformations that belong to different areas of the tICA space revealed that the tICA space spanned by the first two eigenvectors can be divided into the three major regions shown in Fig. 4 which represent, respectively, states with Na^+^ bound to Na2 site, an intermediate state, and the states in which the Na^+^/Na2 had been released.

#### Markov state based kinetic model for the release of Na^+^/Na2

After dimensionality reduction with the tICA method, the next steps in building the MSM include the discretization of the tICA space using a clustering algorithm, and then finding the best number of microstates for MSM construction. As described in Methods section, we used a recently developed MSM scoring method called generalized matrix Rayleigh quotient (GMRQ)^63^, and took the highest GMRQ score to define a set of parameters that result in the best MSM. The GMRQ scores were obtained from the described protocol by randomly selecting half of the simulation trajectories (25 trajectories: 6 in which Na^+^/Na2 releases and 19 in which it doesn’t release) as the test set, and using the other half to build the MSM. The GMRQ scores for all MSMs are shown in Figure S6. We found that the best MSM is obtained by using the *k*-means clustering algorithm to obtain about 100 microstates on the first two tICA eigenvectors (see discussion in Figure S6 caption).

**Figure 5:**
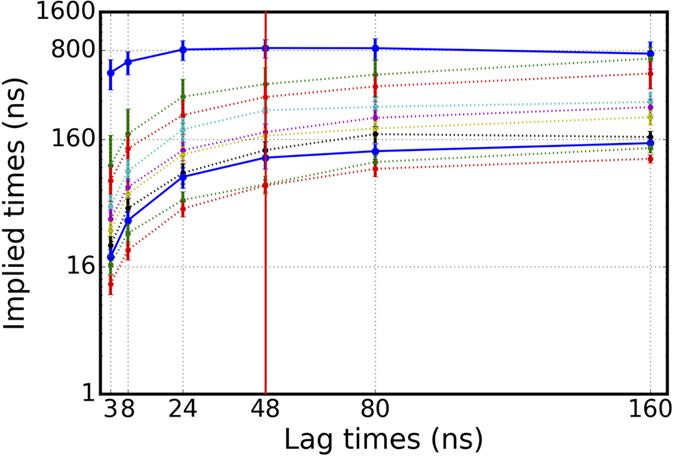
Implied-timescales for the Na^+^/Na2 release kinetics. The Markovian behavior of the system improves as MSM lag-time increases. The 48 ns lag-time (highlighted with red line) was used for constructing transition probability matrix and subsequent analysis. The 1^st^ and 8^th^ relaxation modes are highlighted in bold blue curves. Error bars are calculated using the bootstrap method by using 10 subsamples each generated by randomly choosing 50 trajectories with replacement method yielding between 28 to 36 unique trajectories for each subsample.

Using the best set of parameters identified with the GMRQ analysis, we obtained multiple transition probability matrices (TPMs) in order to construct the *implied time-scales plot* (see Methods). We checked the Markovian behavior of the system by plotting the implied timescales corresponding to the first 10 relaxation modes of each TPM, against multiple lag times (Fig. 5). The final MSM was chosen from the plot by identifying the position at which the curves saturate; a lag-time of ~50 ns is seen to ensure Markovian behavior with minimal loss of data.

**Figure 6:**
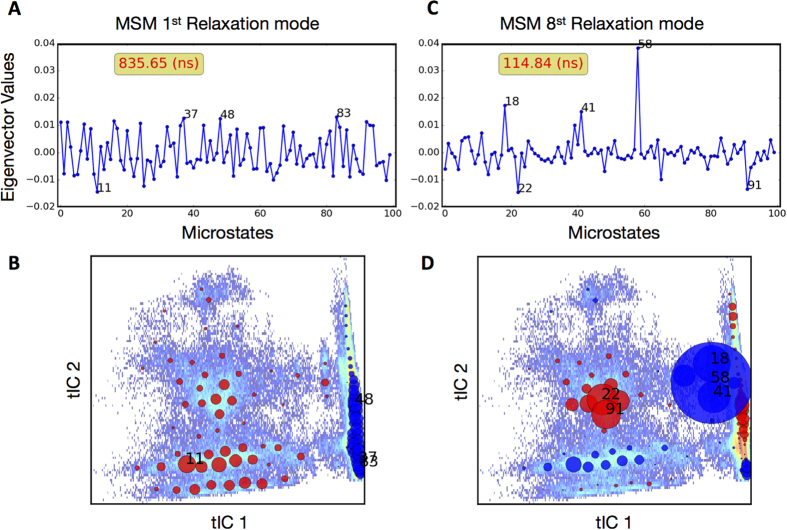
The first and eighth MSM relaxation modes. (**A**) Shows the contribution of each microstate to the first relaxation mode (eigenvector values). The microstates with the largest contributions are numbered. (**B**) The location of each microstate contributing to the first MSM relaxation mode is shown in the space of the first two tICA eigenvectors. Red dots indicate negative values, and blue dots indicate positive values, with the diameters indicating relative contributions. Note that all blue (positive) dots belong to the “Na^+^/Na2 bound” tICA space (cf. Fig. 4), whereas all red (negative) dots belong to the “Na^+^/Na2 released” domain in tICA space. Panels **C** and **D** show the same data as A and B, for the eighth MSM relaxation mode. This relaxation mode corresponds to the intermediate state (see text).

As shown in Fig. 5, the transition probability matrix constructed at 48 ns lag time fulfills the Markovian behavior criterion and this transition probability matrix was chosen for the subsequent analyses. The eigenvector corresponding to the first relaxation mode of the selected transition probability matrix is depicted in Fig. 6A. Negative and positive values correspond, respectively, to states in which Na^+^/Na2 was released, and states in which Na^+^/Na2 was still bound (Fig. 6B). Since the population flows from states with positive values to states with negative values, we conclude that this relaxation mode captures the overall dynamics of the Na^+^/Na2 release process. The Markov model predicts a timescale of about 800 ns for this relaxation mode (Figs 5 and 6). This predicted timescale should be considered as the average time for Na^+^/Na2 release *after* the IC gates are broken, since the ensemble simulations covering the conformational space were initiated from the hDAT conformation in which the IC gates were destabilized due to PIP_2_-mediated N-term/IL4 interactions^28^.

#### Identification of an intermediate state on the release pathway of Na^+^/Na2

**Figure 7:**
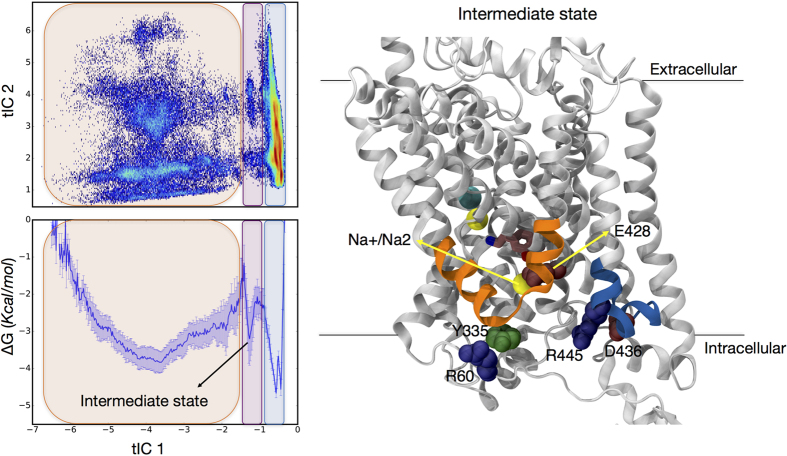
Characteristics of an intermediate state. In the intermediate state the Na^+^/Na2 has left the binding site, is interacting with E428, but has not yet been released to the intracellular solution. *Panels on the left* show the two-dimensional tICA space (upper) and the corresponding free energy profile (lower panel) obtained by taking the natural log of populations based on tICA 1^st^ eigenvector. *The right panel* shows a conformation corresponding to the intermediate state. The R445–E428 interaction is broken and Na^+^/Na2 is stabilized by E428. Color code is the same as in Fig. 1A. Error bars for the free energy values are calculated using the bootstrap method on blocks of frames with 180 ± 70 ns time range that are randomly selected from all the trajectories in which Na^+^/Na2 is released to the intracellular environment.

**Figure 8:**
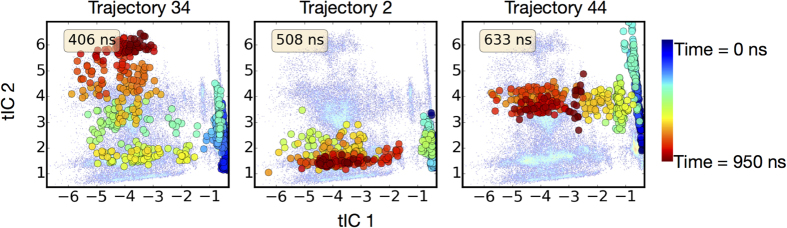
Na^+^/Na2 release pathway on tICA space. Tracking the Na^+^/Na2 release pathway in three representative trajectories (identified on top of each panel) in tICA space. The light blue background shows the tICA landscape obtained from all 50 trajectories. The large colored dots indicate the position of the conformation in tICA space as function of time in the trajectory. The color code of the large dots indicates the time in the trajectory (see color bar). Specifically, the time sequence starts as dark blue color and ends with dark red. Thus, blue dots represent initial states and dark red dots represent final states in each trajectory. The insert in each panel indicates the approximate time where Na^+^/Na2 is released to the intracellular environment. Note that in some trajectories (#44) the metastable intermediate state is well-populated (as seen from the number of yellow and light orange dots in the region of the transition state), while in others (#2) it is not visited. For similar plots for all other trajectories in which Na^+^/Na2 is released, see Figure S7.

For trajectories in which Na^+^/Na2 is released, we calculated the free energy profile of the system based on populations observed along the 1^st^ tICA reaction coordinate (Fig. 7). The profile identifies three major basins, and analysis of conformations belonging to each free energy basin shows that the one with the lowest free energy corresponds to a state where Na^+^ is bound in the Na2 site. The second lowest free energy basin corresponds to a state in which Na^+^/Na2 was released, and the third, middle basin, corresponds to states in which Na^+^/Na2 has left the Na2 site, is coordinated by E428, and therefore has not yet been released to the intracellular solution (Fig. 7). Tracking the Na^+^/Na2 release pathway along the tICA landscape reveals that in some of the release trajectories the metastable intermediate state is more populated than in others in which this state is relatively short-lived (Fig. 8 and Figure S7). The microstates corresponding to the intermediate state (microstates 18, 41, and 58) dominate the relaxation mode that is captured by the 8^th^ MSM eigenvector (Fig. 6C, Figures S8 and S9), which identifies the eighth eigenvector as the most relevant relaxation mode corresponding to this intermediate state; the kinetics of the intermediate state is thus quantified as being on the order of 100 ns (Fig. 6C and D).

Detailed analysis of the trajectories suggests that the intermediate state becomes accessible as a result of disruption of contacts between residues E428 and R445. In Figure S10 the plot of the R445–E428 distance against tIC 1 shows that this interaction is fully broken only in the intermediate state. This establishes the E428/R445 pair as part of the functional IC network (see Fig. 1) that stabilizes hDAT in an inward-closed state as observed in the simulations (see above). Breaking the *ionic lock* between these residues upon PIP_2_-mediated interactions between the N-terminus and IL4, frees E428 to coordinate the Na^+^/Na2 and form the intermediate state.

#### Sodium release pathways

**Figure 9:**
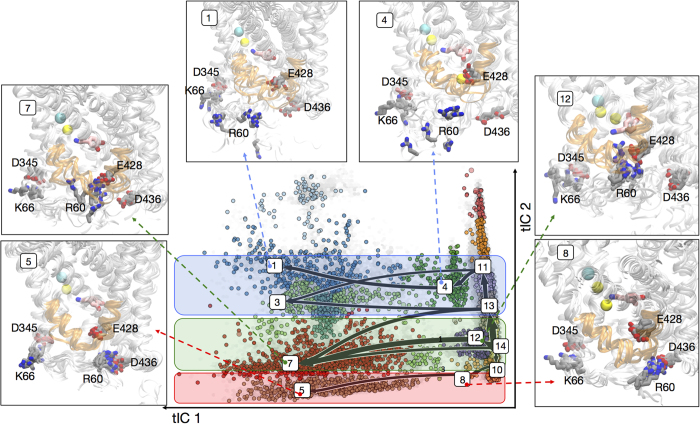
Sodium release pathways. The tICA space (light gray background) is divided into 15 (color coded) macrostates using the PCCA^+^ algorithm, and the most probable release pathways determined with TPT (see text), are indicated on the tICA landscape by arrows that connect macrostates indicated by their numbers. The arrows point from Na^+^-not-released macrostates (on the right) to Na^+^-released macrostates (on the left). The thickness of the arrows indicates the relative magnitude of the flux (i.e., likelihood of release) of the pathway. Flux values are given in Table S3. The tICA spaces corresponding to three distinct pathways (see text) are highlighted in red, green, and blue boxes. Structural representations for key macrostates (numbered) are shown in the structure panels. Residues R60, D436, K66, D345, and E428 are rendered in licorice, IL2 in orange cartoon, dopamine in pink licorice, and the sodium and chloride ions in yellow and cyan spheres, respectively.

The transition path theory (TPT)^64^,^69^ described in the Methods section was used to examine the sodium release pathways and to identify relevant conformational changes associated with each pathway. The analysis is implemented in the MSMBuilder software package^61^ and provides information about the most probable pathways and the overall flux associated with each pathway (see Methods). For TPT analysis, the 100 microstates were grouped into 15 *macrostates* (Fig. 9) based on their kinetic similarity determined with the Robust Perron Cluster Analysis (PCCA^+^) algorithm^66^. Briefly, the PCCA^+^ algorithm lumps microstates into macrostates assuming that microstates with the same signs (positive or negative) in the MSM eigenvectors, will have similar kinetics^70^.

The full transition flux matrix obtained from the model is shown in Figure S12 and error estimates for fluxes are given in Table S3. The top 7 Na^+^/Na2 release pathways identified by the TPT analysis are shown in Fig. 9 (see also Table S4); they contribute >60% of the total Na^+^/Na2 release flux. These pathways could be further clustered into three major sodium release pathways categorized as follows:

The most probable pathway is highlighted in the green box on tICA space in Fig. 9. This pathway is created when the interaction pairs R60-D436 and E428-R445 are replaced by the R60-E428 salt bridge, resulting in the opening of IC gates that allow water penetration as far as the Na2 site. The Na^+^/Na2 is released with a short dwell time in the intermediate state because E428 is engaged in the interaction with R60 and cannot stabilize the Na^+^ ion.

The second major pathway (blue box in Fig. 9) is formed when R60 disengages from D436, and interacts through PIP2 lipids with Lys residues (K257, K260, K264) from intracellular loop 2 (IL2) (Figure S11). These interactions lead to partial unfolding of IL2, a structural feature that has been suggested to be involved in the substrate release mechanism in the related LeuT-fold bacterial MhsT transporter^33^ (see Discussion). In this pathway, Na^+^ is stabilized in the intermediate state through interactions with the available E428.

In the third major pathway (red box in Fig. 9) R60 is engaged with D436, hence the intracellular gate is not fully open. In this pathway the Na2 site is hydrated through a different channel compared to the other two, as the water molecules penetrating the Na2 site reach it through an opening between TM1a and IL3 that results from the breaking of the K66-D345 interaction (cf. Fig. 1B).

It is noteworthy that the TPT analysis of the Markov Models identifies several pathways of release of the Na^*+*^*/Na2 which engage the same set of structural elements but somewhat different configurations and kinetics. These differences depend on specific variations in the mode of interaction of the N-terminus with the intracellular regions of the transporter.*

## Discussion

The role of the Na^+^/Na2 in functional mechanisms of mammalian NSS and bacterial homologs, has been addressed in a variety of studies^16^,^22^,^28^,^30^,^31^. The binding of the Na^+^ ion in the Na2 site was shown in such studies to contribute to the stability of an inward-closed conformation of these transporters, so that release of the Na^+^/Na2 ion is a likely step in the initiation of the inward transport cycle of substrate and sodium. Indeed, we described recently^28^ how the release of the Na^+^/Na2 ion in hDAT is coupled to a spontaneous inward-opening of the transporter in a process triggered by PIP_2_-mediated interactions between the N-terminus and IL4 domains. In particular, these PIP_2_-mediated interactions can disrupt the intracellular network of interactions^24^,^26^ among residues R60, D436, E428, Y335, and R445 that stabilize hDAT in an inward-closed state. Starting from the state in which the intracellular network of interactions is weakened, our Markov model predicts that once the intracellular gates in hDAT are broken, the spontaneous release step of Na^+^/Na2 in the 12 out of 50 replicas of ~1 μs simulations, proceeds relatively fast (timescale of about 800 ns). Indeed, inspection of all 50 trajectories indicates that in longer simulation times the Na^+^/Na2 would have been released in all of them, judging from the accumulation of elements in the dynamic pattern associated with release.

Of the three major release pathways for the Na^+^/Na2 revealed by the TPT analysis, one is seen to go through a metastable intermediate state in which Na^+^/Na2 is coordinated by E428 before its escape (blue box in Fig. 9). We found the average dwell-time of Na^+^/Na2 in this intermediate state to be ~100 ns and showed that the formation of this state is related to disruption of the E428-R445 ionic bond when the N-terminus interacts with intracellular loop 4 (“N-term/IL4 interactions” in Fig. 1B). The E428 side chain can thus participate in both the stabilization of the intracellular gates, and in the coordination of the Na^+^ ion released from the Na2 site. This finding from our analysis is consonant with an experimentally identified functional role of residue E428 in hDAT^71^: the E428Q substitution in hDAT expressed in HEK293 cells was found to impair the binding of the inhibitor CFT, suggesting that this mutation favors an inward-open state in hDAT over the outward-open conformation to which CFT binds. Our computational findings are consistent with these results, as the E428Q substitution will weaken the interaction with R445 and thereby influence the outward-open to inward-open transition equilibrium. This is substantiated as well by available structural data on the bacterial homolog LeuT^4^ in which the Gln that occupies the position aligning with E428 in hDAT, does not interact with the R445-equilvalent residue in LeuT (R375).

It is noteworthy that the E428-dependent intermediate state in the Na^+^/Na2 release pathway we discovered, seems to enable K^+^ ions from the intracellular environment to partition into the Na2 site. The role of K^+^ ions in the function of the related SERT transporter is well-established^67^, and we have recently described significant effects that the presence of a K^+^ ion in the Na2 site can have on the dynamics of the extracellular end of the cognate LeuT and its transport cycle(see refs 72 and 73). While it is tempting to speculate that the ability of K^+^ ions to enter the intracellular region of DAT could facilitate Na^+^/Na2 release from the intermediate state, a more complete evaluation of the impact that K^+^ ions may have on various elements of NSS mechanisms will require future considerations based on quantitative free energy-based computations.

The other major pathway for the Na^+^/Na2 release, enabled when R60 breaks from D436 and engages with Lys residues in the intracellular loop 2 (IL2) through PIP_2_-mediated interactions (Figure S11), is also intriguing as it appears to involve partial unfolding of the IL2 segment. Such a structural perturbation was seen recently in the X-ray structure of related bacterial transporter MhsT^33^, and has been suggested to be a required mechanistic feature of the NSS transporters to allow release of the Na^+^/Na2. Thus, to our knowledge, we provide here the first direct supporting evidence for a dynamic mode of involvement of the IL2 loop unfolding in the Na^+^/Na2 release process.

The third major pathway for the Na^+^/Na2 release is created by water molecules entering the IC vestibule through the region between TM1a and IL3 as a result of the loss of K66-D345 interaction. The involvement of D345 in Na^+^/Na2 release mechanisms identified in this pathway is interesting in light of experimental evidence strongly implicating this residue in the transport mechanisms of hDAT^71^. Specifically, the D345N mutant of hDAT expressed in HEK293 cells has a significantly impaired maximal velocity for dopamine uptake (V_max_) and a reduced maximal capacity of CFT binding (B_max_). Furthermore, these studies concluded that D345N hDAT had a reduced turnover rate, which, in turn, was shown to be largely determined by the rate of reorientation of the unloaded inward-facing transporter. Unlike the wild type Asp at position 345, the Asn would not engage in the same strong interaction with K66 and therefore, according to our computational results, would increase the probability for opening the Na^+^/Na2 release pathway. Since the release concomitantly widens the intracellular vestibule, the impairment of the K66-D345 interaction is then predicted to affect the kinetics of inward-open to inward-close transition, consistent with the conclusions from the cited experiments.

The detailed structural rearrangements and membrane-supported interactions underlying the kinetic model for Na^+^/Na2 release after the intracellular gates are broken due to the PIP_2_-mediated N-term/IL4 association, represent steps in an allosteric mechanism of a key mechanistic stage in the transport cycle of NSS transporters^36^,^74^. We showed that this allosteric mechanism could follow distinct transition paths in which the same set of structural elements are engaged, but with somewhat different configurations of interactions. Thus, the different modes in which the N-terminus can interact with the intracellular region of the transporter, and variations in the manner in which E428 can stabilize different salt bridges and thereby modulate the mode of Na^+^/Na2 release, can result in different kinetic pathways even for the relatively simple step of Na^+^/Na2 release in the transport cycle of hDAT. Since these pathways were shown in our analysis to correspond to different energy basins, it is clearly desirable to develop a comprehensive kinetic model of the entire process, including the intracellular gate-breaking step, and to base the mechanistic analysis of the transition from the outward-facing configuration to the inward-facing configuration of the transporter on similar considerations of the energetics. Further studies are now ongoing to collect such information.

## Additional Information

**How to cite this article:** Razavi, A. M. *et al*. A Markov State-based Quantitative Kinetic Model of Sodium Release from the Dopamine Transporter. *Sci. Rep.* **6**, 40076; doi: 10.1038/srep40076 (2016).

**Publisher's note:** Springer Nature remains neutral with regard to jurisdictional claims in published maps and institutional affiliations.

## Supplementary Material

Supplementary Information

## Figures and Tables

**Table 1:** Lipid composition* of the model membrane used in this study.

LIPID	Extracellular	Intracellular	Total
Cholesterol	29	24	53
POPC	125	26	151
POPE	0	92	92
POPS	0	20	20
PI(4,5)P_2_	0	18	18
SM	12	0	12
Number of lipids	166	180	346

^*^The lipid abbreviations are as follows: POPC – 1-palmitoyl-2-oleoyl-sn-glycero-3-phosphocholine, POPE – 1-palmitoyl-2-oleoyl-sn-glycero-3-phosphoethanolamine, POPS – 1-palmitoyl-2-oleoyl-sn-glycero-3-phosphoserine, PI(4,5)P_2_- phosphatidylinositol-*4*,*5*-bisphosphate, SM-sphingomyelin.

**Table 2:** Parameters used for dimensionality reduction and tICA construction.

Parameters reflecting Na^+^ motion	Parameters related to water penetration
Na^+^/Na2 to Na^+^/Na1 distance	R60–Y335 distance
Na^+^/Na2 to E428 distance	R60–E446 distance
Na^+^/Na2 to D421 distance	R60–E428 distance
Na^+^/Na2 to D79 distance	R60-D436 distance
	Y335–E428 distance
	D436–R445 distance
	E428–R445 distance
	Na^+^/Na2 water coordination number
