# Supplementary material for: A Markov State-based Quantitative Kinetic Model of Sodium Release from the Dopamine Transporter
Source: Sci Rep. 2017 Jan 6;7:40076. doi: 10.1038/srep40076 (PMC5216462; doi:10.1038/srep40076)
Supplement: Supplementary Information [file srep40076-s1.pdf]

***A Markov State-based Quantitative Kinetic Model of Sodium Release from the Dopamine Transporter***

Asghar M. Razavi[1], George Khelashvili[1], Harel Weinstein[1,2]*

[1]Department of Physiology and Biophysics, Weill Cornell Medical College of Cornell University, New York, NY, 10065, USA. [2]Institute for Computational Biomedicine, Weill Medical College of Cornell University, New York, NY 10065, USA. *Corresponding author: (email: haw2002@med.cornell.edu)

## Supplementary Information

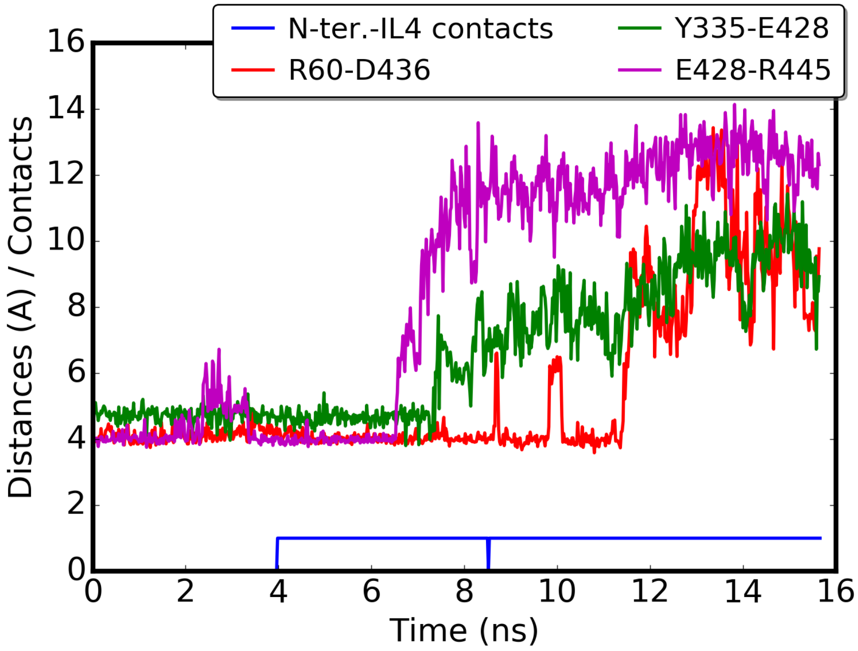


**Figure S1**. Intracellular gate opening during the equilibration phase. As the $PIP_2$ mediated interaction of the N-terminus (R51 here) and IL4 (R443) is established, the network of interactions at the intracellular gate (R60–D436, Y335–E428, and R445–E428) is destabilized.

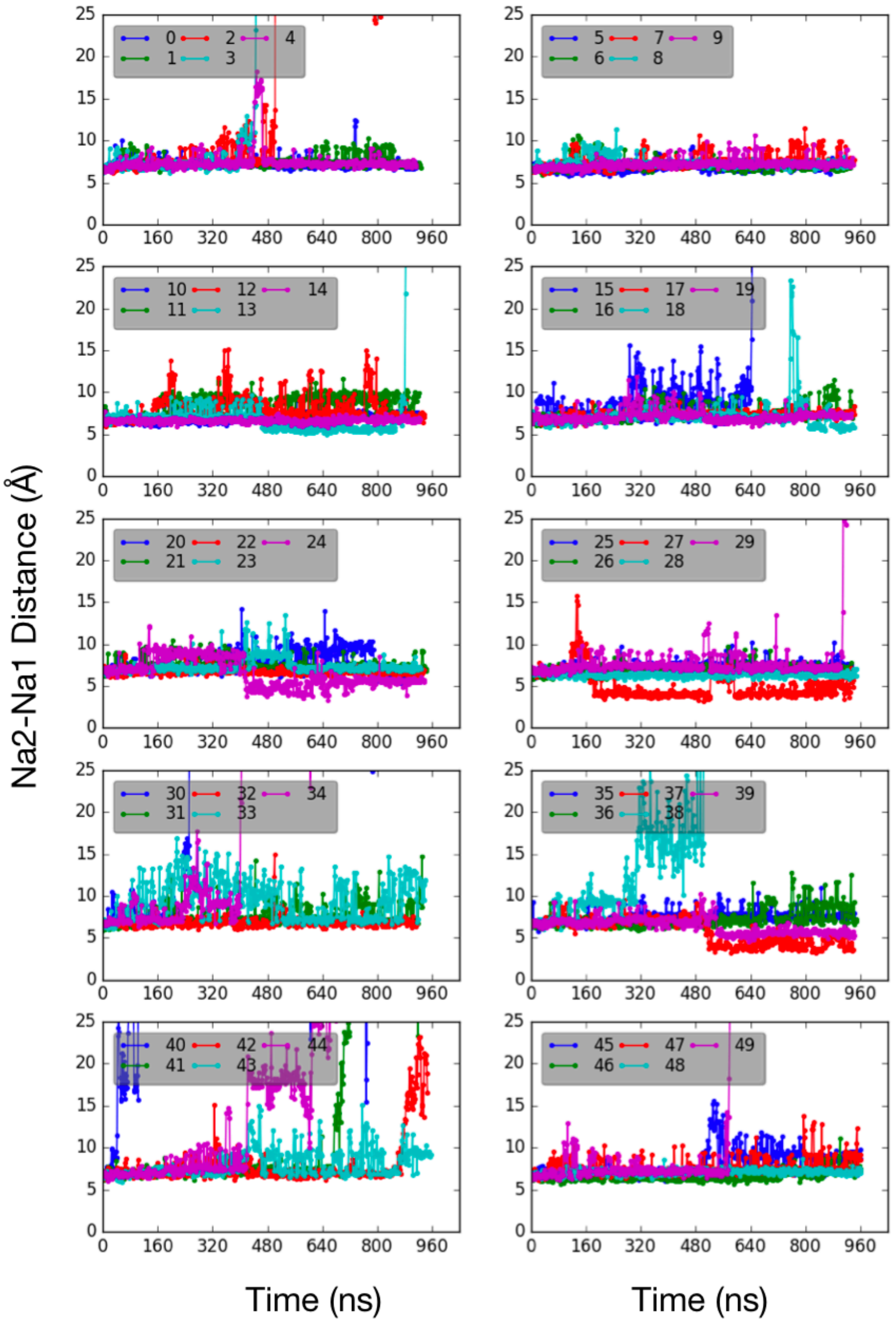


**Figure S2**. Time evolution of the Na1-Na2 distance in all 50 MD trajectories. Na+/Na2 release into intracellular solution (distance beyond 25Å) was observed in 12 trajectories.

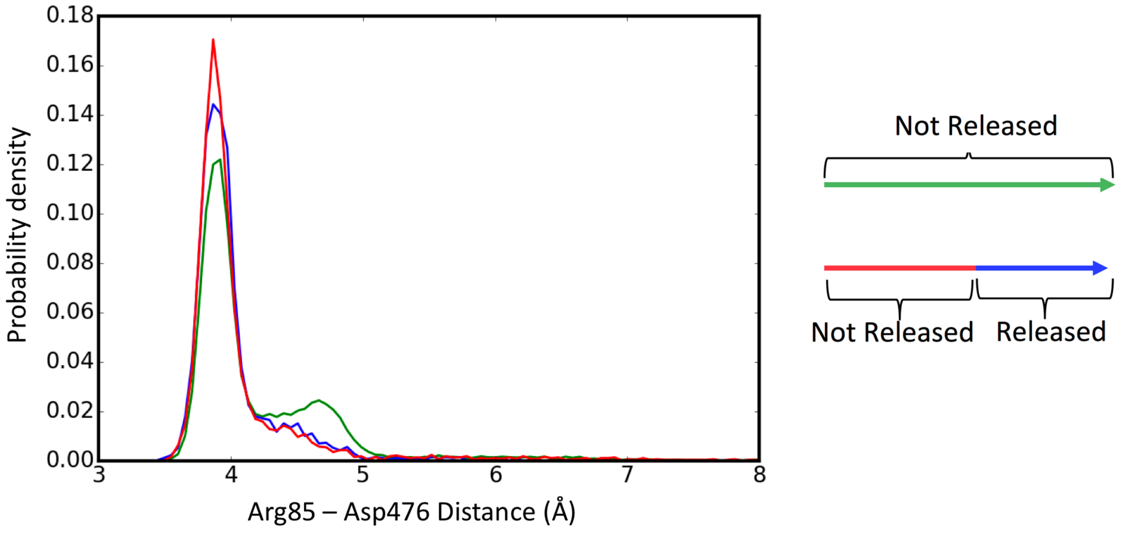


**Figure S3**. Extracellular gate dynamics represented by the Arg85 and Asp476 distance measured between terminal carbon atoms in both Arg and Asp. In trajectories that Na+/Na2 releases to intracellular (red and blue curves) the extracellular gate is more closed than trajectories that Na+/Na2 doesn't release to intracellular (green curve). For a detailed description of classifying trajectories in red, blue, and green curves see the caption of Figure 2 in the main text.

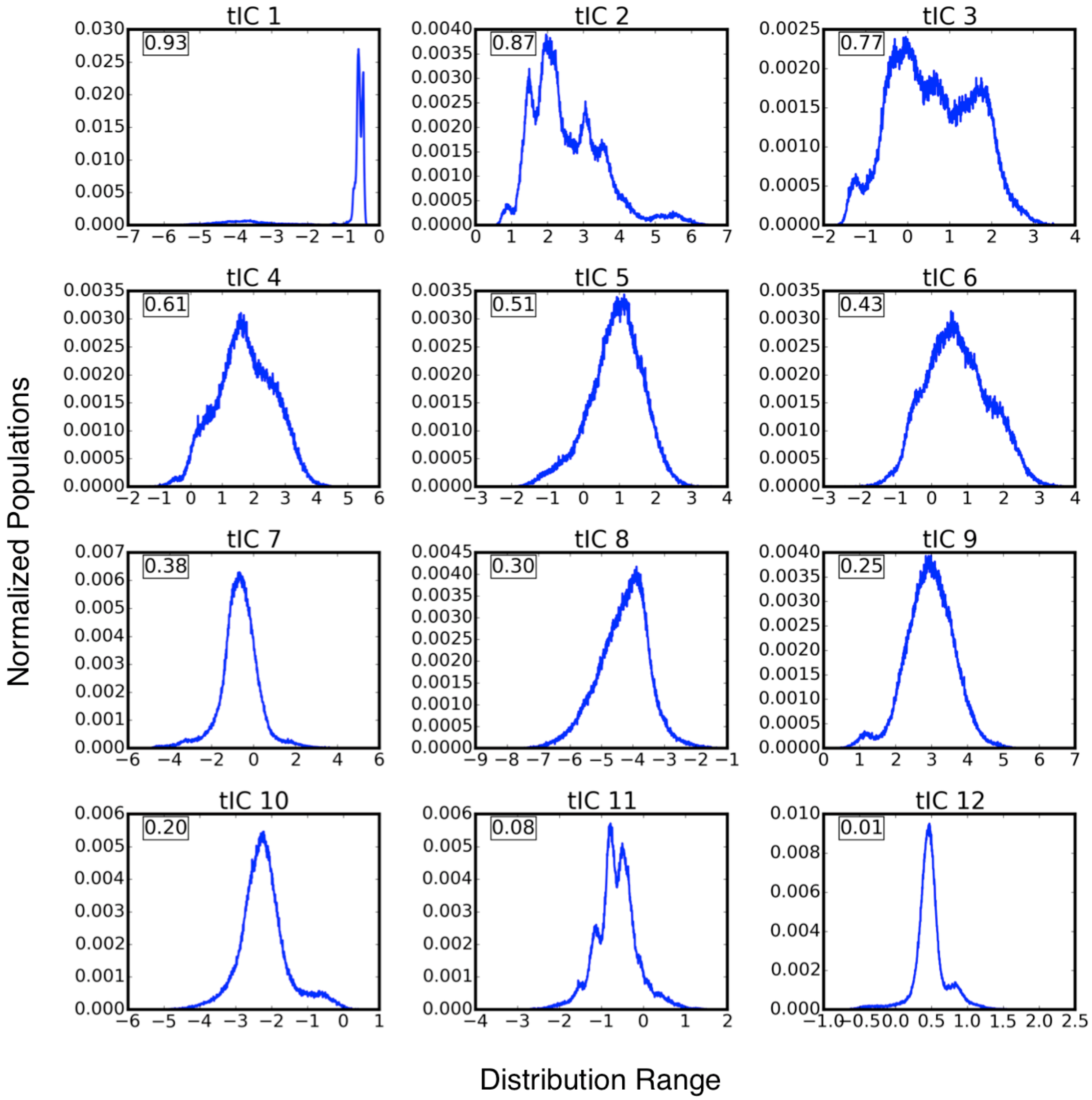


**Figure S4**. Distributions of simulation data projected on all 12 tICA eigenvectors. Only the first three eigenvectors produce a clear non-Gaussian distribution, and thus can be used for conformational space discretization. The insets show the corresponding eigenvalue for each eigenvector.

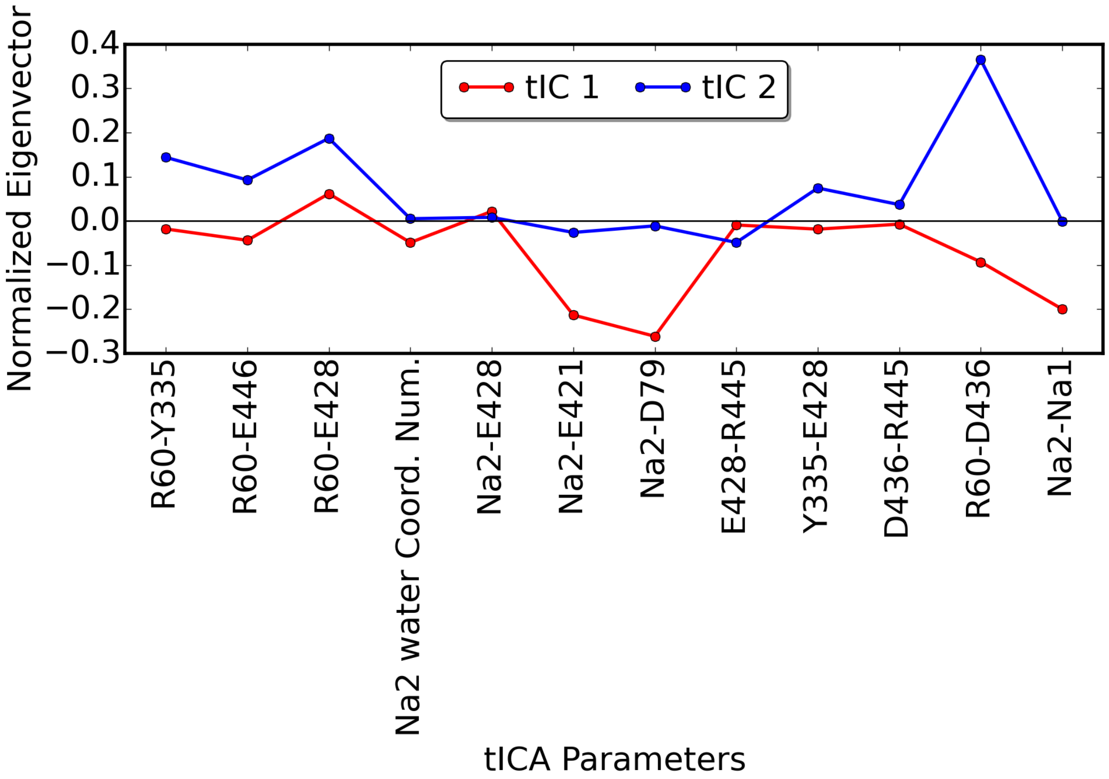


**Figure S5**. Contribution of tICA parameters to the first two eigenvectors. Parameters reflecting $Na^+$ motion contribute more to tIC 1; parameters reflecting water penetration have higher contributions in tIC 2.

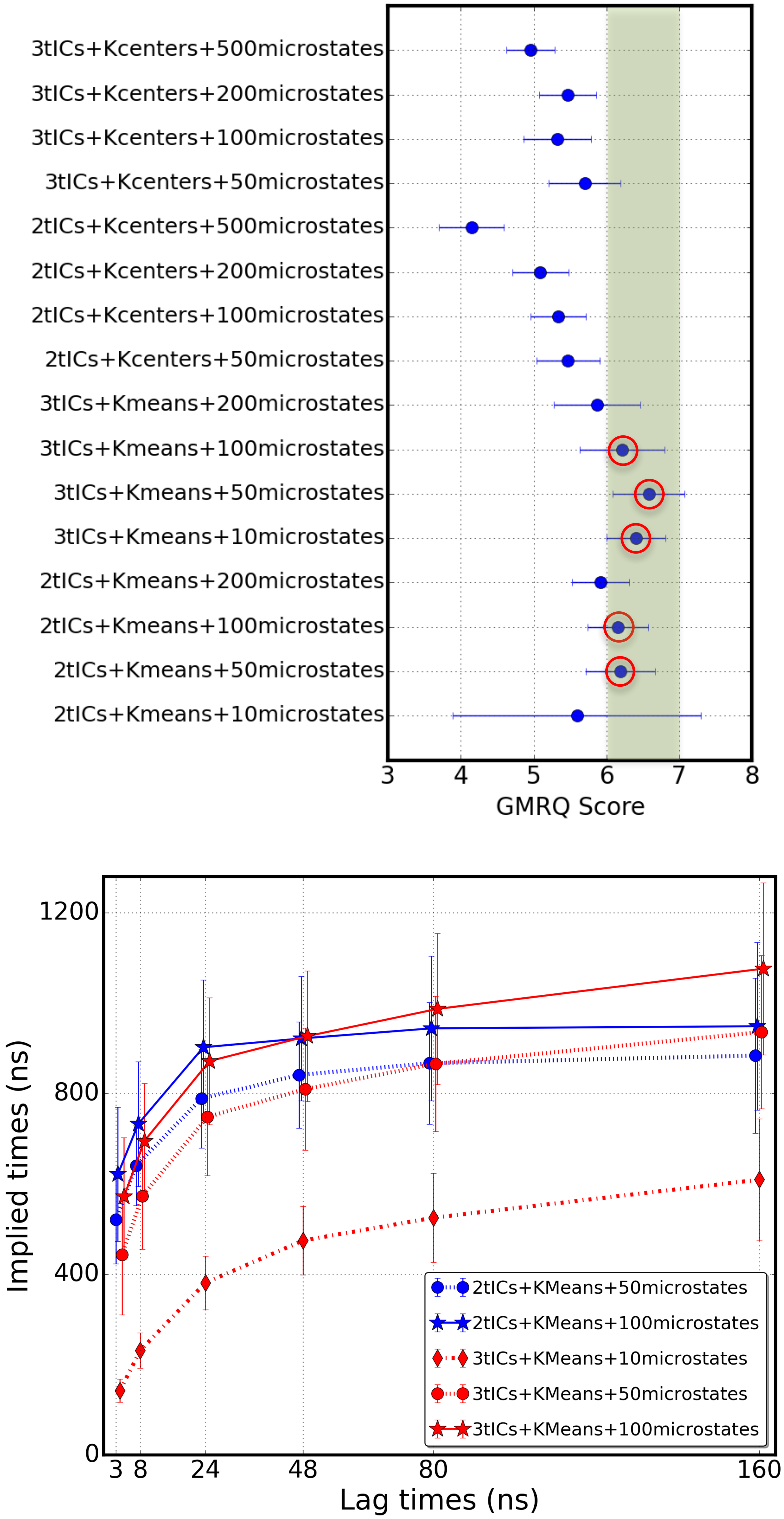


**Figure S6**. *Top panel:* GMRQ score for MSM parameters. The highlighted shaded area indicates the (higher) scores of models that are statistically superior to others. For each set of parameters (identified by a name describing the number of tICA

eigenvectors + the clustering algorithm + the number of microstates), the mean and error bar are the result of 200 GMRQ scores calculated by randomly dividing the data in two sets: a training set and a test set (see Methods). The first 10 MSM eigenvectors obtained with a lag time of 48 ns are used (as described in Methods) for all models. The *k*-means clustering algorithm is consistently performing better than the *k*-centers algorithm in this task. The models with highlighted red circles were subjected to further analysis based on implied time-scales plots. *Lower panel:* The first implied timescale calculated for each of the models highlighted with red circles in the top panel. The MSMs with 2tICs (blue curves) act Markovian shortly after about 24 ns lag time. On the other hand, MSMs with 3tIC s (red curves) don't satisfy Markovian criterion even after 80 ns. Hence, the MSMs with 2tICs are considered superior to MSMs with 3tICs. Since MSMs always underestimate relaxation timescales (due to discretization of the conformational landscape) the model with the slower implied time (2tICs+KMeans+100microstae) is preferable to the other one (2tICs+KMeans+50microstates). This model was chosen for all subsequent analyses presented in the main text. Error bars are calculated using a bootstrap method with randomly selecting 50 trajectories and calculating implied timescales and repeating the process 1000 times for each model. As the lag time increases, the error bars also increase due to the increase in finite sampling errors.

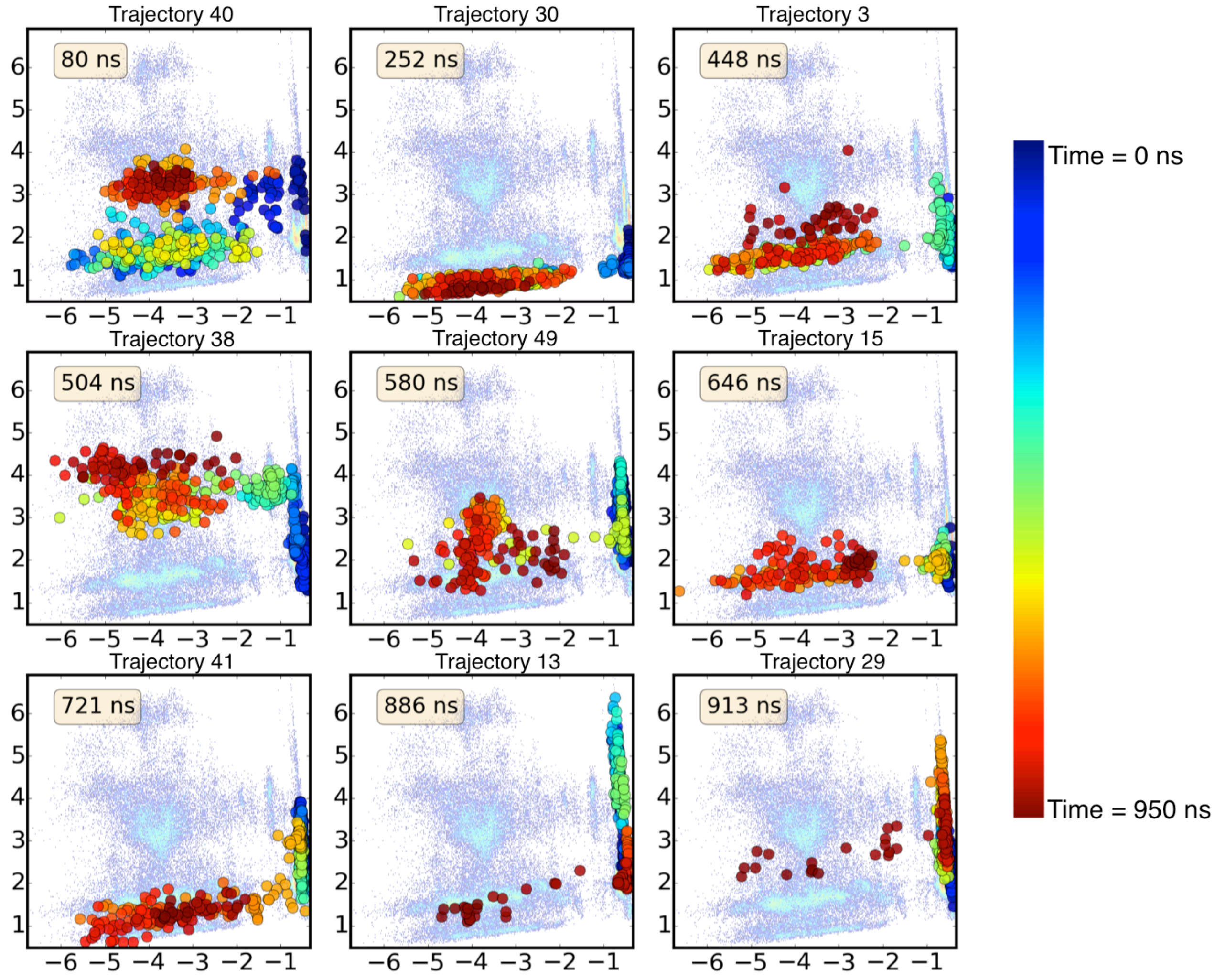


**Figure S7.** Time evolution of the trajectories where Na+/Na2 is released at different times (trajectory lengths). For each release time to the intracellular environment, (shown as insert), the colored dots indicate the time evolution (from blue to red) for the trajectories that released at that time, mapped on the first two tICA coordinates (cf. Fig 8 in main text).

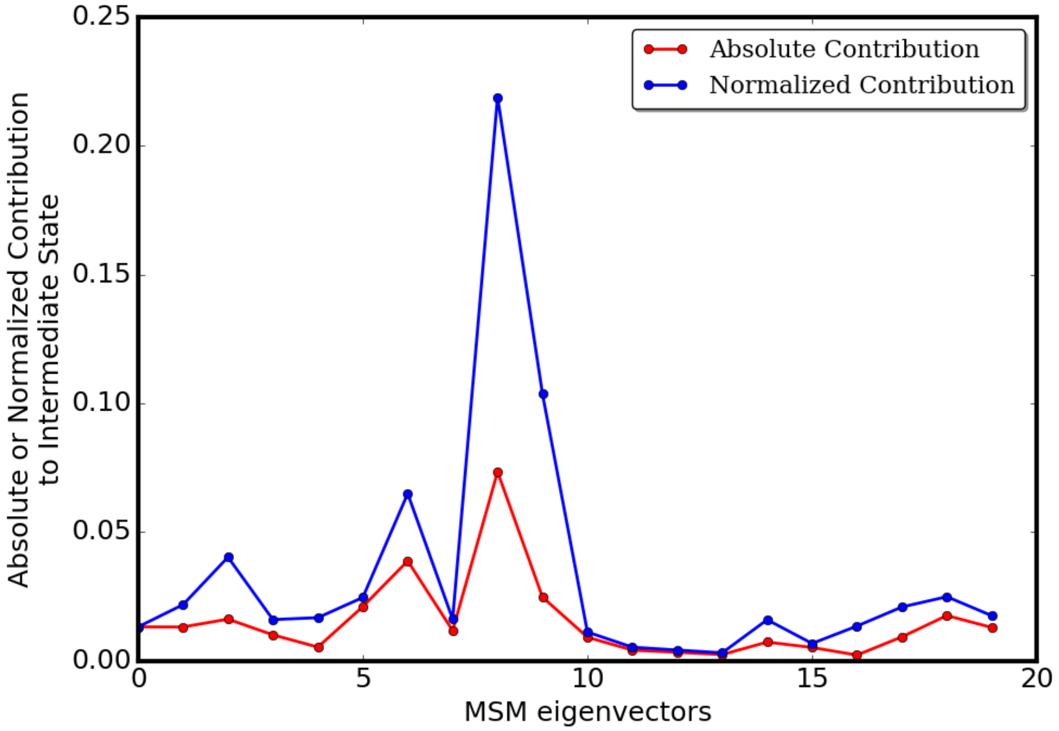


**Figure S8.** Identification of the MSM relaxation mode for the intermediate state. The total contribution of each eigenvector to the intermediate state is shown as both absolute ($\sum_i abs(v_i)$) and normalized ($\sum_i \frac{abs(v_i)}{\sum_{all} v}$) values; $v_i$ are the eigenvectors corresponding to intermediate state microstates.

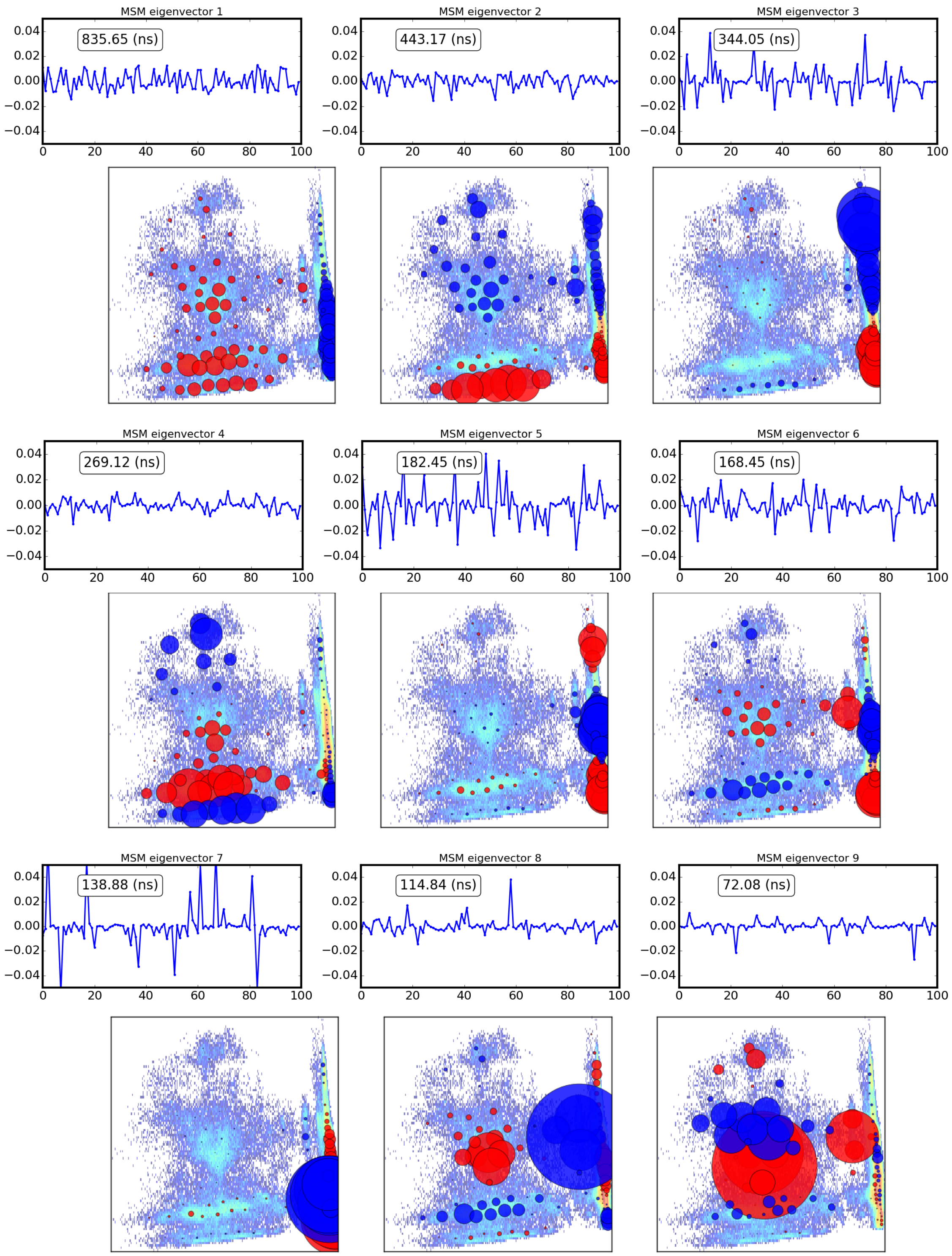


**Figure S9.** Markov State Model eigenvectors and corresponding relaxation modes on tICA landscapes (cf. Figure 6 in main text). Colored circles identify the center of the microstate into which the trajectory snapshots are clustered. Red and blue colors represent negative and positive eigenvectors, respectively, and the size of the

circles is proportional to eigenvectors value. The relaxation times are also shown. The MSM 8th eigenvector contributes most to the intermediate state.

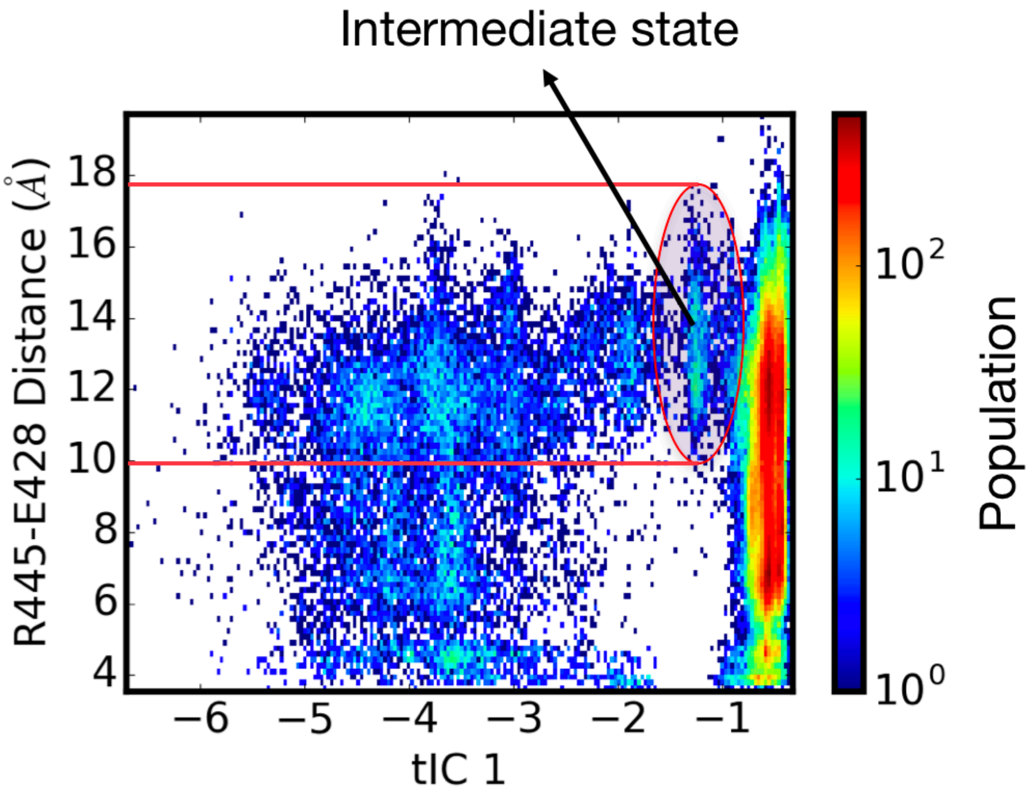


**Figure S10**. The population weighted 2D histogram of R445–E428 distance against tIC 1. The oval highlights the intermediate state and lines show R445–E428 distance range in Å.

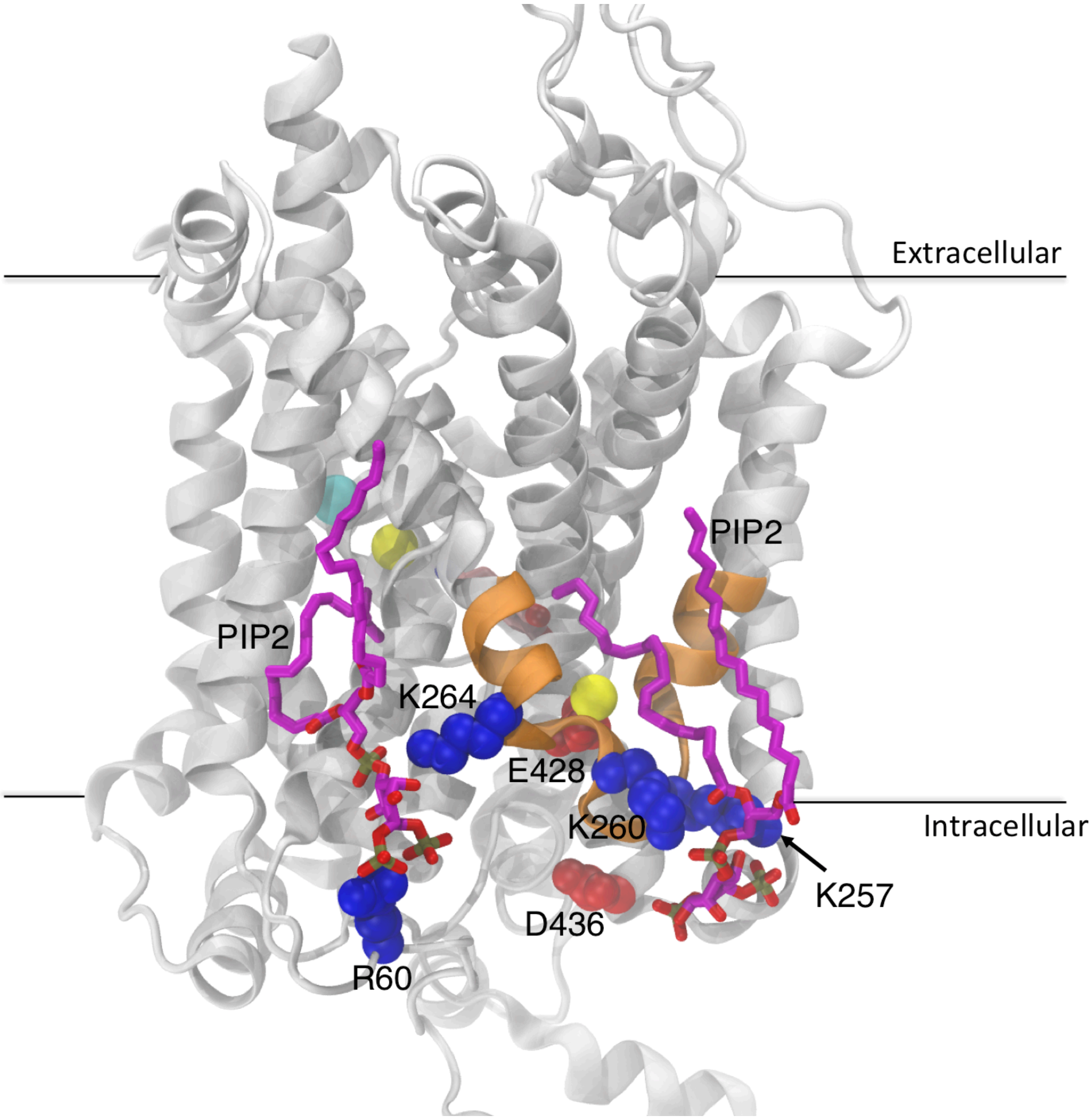


**Figure S11**. $PIP_2$ mediated N-terminus interactions with IL2. R60 from the N-terminus is interacting with K264 from IL2 (orange cartoon) through a $PIP_2$ lipid (in magenta). Other Lys residues from IL2 (K257 and K260) are interacting with $PIP_2$ as well. These interactions result in partial unfolding of IL2. Na+/Na2 is interacting with E428. Color code is the same as in Figure 1A**.**

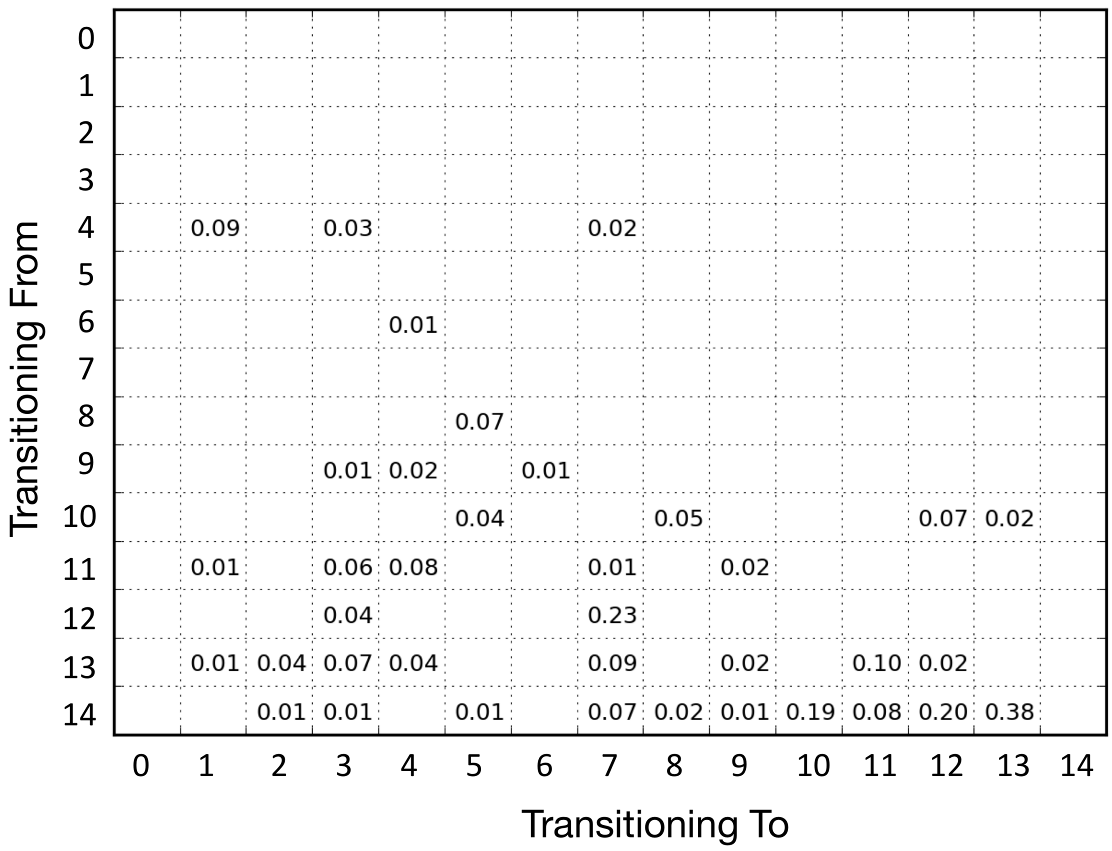


**Figure S12.** The transition flux matrix presents the flux values for transitions from macrostates numbered on the vertical axis to those identified on the horizontal axis. Only transition values of 0.01 and higher are shown in the matrix. Error estimates for these transitions are given in Table S3.

**Table S1.** Correlation values for water coordination number of Na+/Na2 and distance between Na+/Na2 and sodium at Na1 site. The correlation numbers are calculated by considering all frames up to the time when Na+/Na2 is released to the intracellular environment.

| Trajectory number | Absolute Na+/Na2 release time (ns) | Correlation between Na2–Na1 distance and water coordination number of Na+/Na2 |
|---|---|---|
| 2 | 504 | 0.76 |
| 3 | 448 | 0.70 |
| 13 | 886 | 0.56 |
| 15 | 646 | 0.38 |
| 29 | 913 | 0.41 |
| 30 | 252 | 0.74 |
| 34 | 406 | 0.60 |
| 38 | 504 | 0.75 |
| 40 | 80 | 0.78 |
| 41 | 721 | 0.50 |
| 44 | 633 | 0.62 |
| 49 | 580 | 0.44 |
| Average correlation for the 12 trajectories: 0.60 | | |

**Table S2.** Confidence levels (at 68% and 95%) for water distribution in the extracellular cavity, obtained by assuming normal distributions.

| Trajectories | | 68 percent confidence interval | 95 percent confidence interval |
|---|---|---|---|
| Na+/Na2 is **Not Released** | | 25.7 – 38.7 | 19.4 – 45.0 |
| Na+/Na2 is **Released** | before the release | 22.9 – 35.2 | 17.0 – 41.2 |
| | after the release | 23.5 – 35.0 | 17.9 – 40.6 |

**Table S3**. Absolute values and standard deviations of fluxes calculated for all transitions (only fluxes >0.01 are listed). Standard deviations are calculated using the bootstrap method with 10 subsamples selected randomly from 40 individual trajectories out of the total 50 trajectories.

| Macrostate Transition | Flux |
|---|---|
| 4 --> 1 | 0.094 ± 0.067 |
| 11 --> 1 | 0.013 ± 0.006 |
| 13 --> 1 | 0.010 ± 0.008 |
| 13 --> 2 | 0.043 ± 0.040 |
| 14 --> 2 | 0.014 ± 0.012 |
| 4 --> 3 | 0.035 ± 0.023 |
| 9 --> 3 | 0.013 ± 0.010 |
| 11 --> 3 | 0.056 ± 0.026 |
| 12 --> 3 | 0.036 ± 0.014 |
| 13 --> 3 | 0.074 ± 0.034 |
| 14 --> 3 | 0.011 ± 0.008 |
| 6 --> 4 | 0.014 ± 0.006 |
| 9 --> 4 | 0.016 ± 0.008 |
| 11 --> 4 | 0.079 ± 0.068 |
| 13 --> 4 | 0.041 ± 0.018 |
| 8 --> 5 | 0.066 ± 0.072 |
| 10 --> 5 | 0.037 ± 0.047 |
| 14 --> 5 | 0.014 ± 0.012 |
| 9 --> 6 | 0.011 ± 0.005 |
| 4 --> 7 | 0.019 ± 0.011 |
| 11 --> 7 | 0.013 ± 0.005 |
| 12 --> 7 | 0.232 ± 0.094 |
| 13 --> 7 | 0.094 ± 0.023 |
| 14 --> 7 | 0.074 ± 0.043 |
| 10 --> 8 | 0.051 ± 0.053 |
| 14 --> 8 | 0.025 ± 0.012 |
| 11 --> 9 | 0.017 ± 0.011 |
| 13 --> 9 | 0.016 ± 0.008 |
| 14 --> 9 | 0.012 ± 0.007 |
| 14 --> 10 | 0.189 ± 0.069 |
| 13 --> 11 | 0.097 ± 0.055 |
| 14 --> 11 | 0.082 ± 0.018 |
| 10 --> 12 | 0.068 ± 0.039 |
| 13 --> 12 | 0.023 ± 0.022 |
| 14 --> 12 | 0.197 ± 0.034 |
| 10 --> 13 | 0.022 ± 0.014 |
| 14 --> 13 | 0.377 ± 0.046 |

**Table S4**. MSM-predicted sodium release pathways and fluxes.

| Pathway | Normalized Flux | Accumulated Flux |
|---|---|---|
| [14 12  7] | 0.20 | 0.20 |
| [14 13  7] | 0.10 | 0.30 |
| [14 13 11  4  1] | 0.08 | 0.38 |
| [14  7] | 0.07 | 0.45 |
| [14 13  3] | 0.07 | 0.52 |
| [14 11  3] | 0.06 | 0.58 |
| [14 10  8  5] | 0.05 | **0.63** |
